# Fluorescent nanodiamond labels: Size and concentration matters for sperm cell viability

**DOI:** 10.1016/j.mtbio.2023.100629

**Published:** 2023-04-10

**Authors:** Claudia Reyes San-Martin, Yue Zhang, Thamir Hamoh, Lotte Berendse, Carline Klijn, Runrun Li, Alina Sigaeva, Jakub Kawałko, Hui Ting Li, Jian Tehrani, Aldona Mzyk, Romana Schirhagl

**Affiliations:** a Groningen University, University Medical Center Groningen, Antonius Deusinglaan 1, 9713 AW, Groningen, Netherlands; b AGH University of Science and Technology, Academic Centre for Materials and Nanotechnology, Al. A. Mickiewicza 30, 30-059, Krakow, Poland; c Department of Obstetrics and Gynaecology, University of Groningen, University Medical Centre Groningen, 9700 RB, Groningen, Netherlands; d Institute of Metallurgy and Materials Science, Polish Academy of Sciences, Reymonta 25, 30-059, Krakow, Poland

**Keywords:** Nanodiamonds, NV centers, Biocompatibility, Sperm cells

## Abstract

Nanodiamonds are increasingly popular in biomedical applications, including optical labelling, drug delivery and nanoscale sensing. Potential new applications are in studying infertility or labelling sperm cells. However, for these applications, it is necessary that nanodiamonds are inert and do not alter sperm properties. In this article, we assessed the biocompatibility of nanodiamonds in detail. We investigated different sizes and concentrations of nanodiamonds and sperm preparation methods. We evaluated if the metabolic activity, membrane integrity, morphology and formation of reactive oxygen species were altered. These parameters were tested for sperm cells in their uncapacitated and capacitated states. Unfortunately, FNDs are not universally biocompatible. Generally, cells in the capacitated state are more prone to stress. Additionally, larger particles and lower concentrations are tolerated better than smaller and higher concentrated particles.

## Graphical abstract

**Figure:**
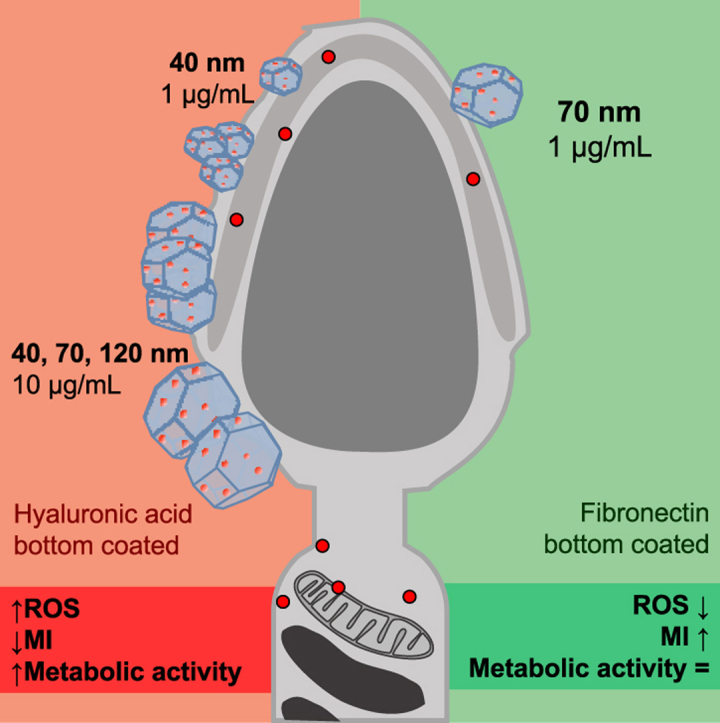

## 1 Introduction

Infertility is currently affecting 15% of couples worldwide [1]. Due to lifestyle changes, increasing paternal age, and environmental factors, male fertility issues are expected to increase. The distinctive properties of nanoparticles (NPs) make them potent tools for assisted reproductive techniques (ART). So far, NPs have been used as part of hybrid micromotors to restore sperm motility and sperm separation and labelling [2].

Barchanski et al. have labelled sperm cells with bio-conjugated gold NPs [3,4]. Nevertheless, researchers observed that gold NPs had an adverse effect on semen quality [5]. Similarly, other metal particles (e.g. silver, zinc oxide and nickel NPs) have been regarded as triggers of excessive reactive oxygen species (ROS) production, which decreased sperm viability [6,7]. Vasquez et al. have developed bioluminescent magnetic nanoparticles as potential probes for imaging and tracking spermatozoa [8]. Researchers have reported that iron oxide NPs coated with lectins enable efficient selection of the most vital spermatozoa in semen [9]. These NPs seem to be biocompatible with spermatozoa.

Besides sperm separation and labelling, nanoparticles have been of interest as nanocarriers for improving transgenesis and targeted delivery of molecules. It has been shown that mesoporous silica NPs loaded with fluorescent nucleic acid or a fluorescent protein are promising candidates for molecule delivery into male gametes [10]. However, further studies by Ren et al. have provided evidence that silica nanoparticles induce reversible damage to spermatogenic cells [11]. On the other hand, Wang et al. have pointed out that spermatozoa could be affected by a magnetic field which is often applied in combination with magnetic NPs [11]. These findings should raise awareness of the opportunities and risks associated with commonly used nanoparticles.

Commonly used in the field, nanoparticles, despite their many benefits, have several disadvantages. One of the most important of these disadvantages is potential toxicity. Particularly interesting nanoparticles that might be useful for reproductive biology and ART are fluorescent nanodiamonds (FNDs). FNDs are increasingly popular as fluorescent labels for their unprecedented photostability. FNDs have been used for the long-term tracking of vesicles or entire cells [12]. Furthermore, they are promising for biosensing applications since their fluorescence changes depending on their magnetic surroundings [13]. It has been shown that FNDs could be applied to detect free radical generation in yeast [14] and macrophages [15]. The surface chemistry of FNDs may be modified to be used as carriers for drug delivery or in transgenesis [[16], [17], [18], [19], [20]]. FNDs are generally assumed to have excellent biocompatibility, which has, for instance, been shown for neurons, human lung epithelial cells, liver cells, and HeLa cells, as well as in several in-vivo models [21,22,23,24,25]. However, in some cases, low cytotoxicity (especially at high doses) or decreased proliferation have been observed [26,27,28]. How FNDs interact with sperm cells under differential maturity stages (uncapacitated and capacitated) and if they influence their properties is crucial and, so far, unknown. Herein, we have evaluated the biocompatibility of FNDs to sperm cells for the first time and showed their potential as fluorescent labels.

## 2 Materials and methods

### 2.1 Diamond materials

Fluorescent nanodiamonds (FNDs) with a hydrodynamic diameter of 40 ​nm, 70 ​nm and 120 ​nm were provided by Adámas Nano (Raleigh, NC, USA). In this study, we have used oxygen-terminated nanodiamonds as received. The manufacturer produces them by grinding high-pressure high-temperature (HPHT) diamonds. These nanodiamonds contain fluorescent NV centres naturally. However, their concentration is very low. To increase the number of defects and thus the brightness, the FNDs were irradiated with an electron beam at 3 ​MeV at a fluence of 5∗10^19^ e/cm^2^. During this process, vacancies and nitrogen centres are created separately. During high-temperature annealing above 600 ​°C under vacuum for 2 ​h, vacancies become mobile and form NV centres with the nitrogen atoms [29]. Our previous studies have characterised the shape and surface chemistry of FNDs [30]. The particle size is confirmed in Figs. S1 and a chemical characterisation via infrared spectroscopy is shown in Fig. S5.

### 2.2 Sperm selection

The boar semen was obtained from the Varkens KI Nederland BV. Semen was centrifuged (300 ​g, 20 ​min) over a density gradient of Ficoll-400 (Sigma Aldrich, The Netherlands), separating spermatozoa by their motility. The density gradient of Ficoll-400 was obtained with a 45% (v/v) density top layer and 80% (v/v) density lower layer. The motile spermatozoa formed a soft pellet at the bottom of the centrifugation tube. Cells were collected, rinsed with Ringer solution and spun down (400 ​g, 10 ​min). After centrifugation, the pellet was dispersed in the uncapacitating (modified Human Tubal Fluid medium; mHTF - without albumin, bicarbonates, calcium) or capacitating medium. The capacitating medium (complete Human Tubal Fluid-HTF-) consists of NaCl 101.6 ​mM, KCl 4.69 ​mM, glucose 2.78 ​mM, KH_2_PO_4_ 0.37 ​mM, MgSO_4_ 0.2 ​mM, sodium lactate 21.4 ​mM, sodium pyruvate 0.33 ​mM, BSA 4 ​mg/ml, NaHCO_3_ 25 ​mM, CaCl_2_ 2.04 ​mM) [31] or uncapacitating medium (modified Human Tubal Fluid -mHTF-, without BSA, NaHCO_3_, and CaCl_2_) [32].).

### 2.3 Sperm immobilisation and incubation with FNDs

Glass-bottom dishes modified with 5 ​μg/ml fibronectin (Sigma Aldrich, The Netherlands) or 1 ​mg/ml hyaluronic acid (LifeCore, USA) were used to immobilise spermatozoa (1.5 ​× ​10^4^ ​cells/cm^2^). Cells were incubated for 30 ​min at 37 ​°C to slow down their movement. Then sperm cells were treated with the suspension of fluorescent nanodiamonds of various sizes (40 ​nm, 70 ​nm or 120 ​nm) in the mHTF or HTF medium at concentrations of 1, 5, 10 or 20 ​μg/ml and kept at 37 ​°C for 4 ​h. Afterwards, the excess nanodiamonds were removed by rinsing samples with the fresh medium.

### 2.4 Characterisation of FNDs

The average hydrodynamic diameter of 1 ​μg/mL FNDs (40 ​nm, 70 ​nm or 120 ​nm) in mHTF or HTF medium was evaluated by dynamic light scattering (DLS) at 25 ​°C. The tests were performed by Zetasizer Nano ZS ZEN3500 (Malvern) equipped with a 633 ​nm He–Ne laser using back-scattering detection. Similar to previous work, FNDs in H_2_O were used for comparison to see if aggregation occurs [[33], [34], [35]].

### 2.5 Labelling sperm cells containing FNDs

Labelling of sperm cells with fluorescent nanodiamonds was performed before and after capacitation. Samples were fixed in 3.7% PFA (Paraformaldehyde) and stained with 2 ​μg/mL phalloidin-FITC (Sigma-Aldrich, The Netherlands) to label F-actin and 4 ​μg/mL DAPI to visualise the nucleus (Sigma-Aldrich, The Netherlands). The specimens were imaged using 405 ​nm, 488 ​nm and 561 ​nm lasers of the LSM780 confocal microscope. Images were analysed by FIJI 2.0.0 software. The location of FND was investigated with scanning electron microscopy (SEM) using an FEI Versa 3D FEG with an EDT secondary electron detector. An acceleration voltage of 10 ​kV and a beam current of 4 ​nA were used. Before the SEM observations, a 20 ​nm layer of gold was sputtered onto the samples using Leica EM ACE600. The purpose of this coating is to increase the surface conductivity of the sample and avoid charging artefacts.

### 2.6 Metabolic activity of FNDs-treated sperm cells

The MTT assay was performed to determine the metabolic activity of sperm cells exposed to the fluorescent nanodiamonds. Cells cultured in a 24-well plate were treated with 0.75 ​μg/ml thiazolyl blue tetrazolium bromide (MTT) dissolved in the mHTF or HTF medium. Sperm cells treated with 0.1 ​M HCl were used as a positive control. Samples were incubated for 3 ​h at 37 ​°C. Then the reagent was removed, and 2-propanol was added to dissolve the formazan formed inside the cells. The absorbance of the obtained coloured solution was measured using a FLUOstar Omega Microplate Reader (BMG Labtech, The Netherlands) at 570 ​nm. The results from three independent repetitions were normalised over the negative control (cells untreated with FNDs) after subtraction of the background (medium without cells).

### 2.7 Effect of FNDs on sperm membrane integrity

Plasma membrane integrity was determined using a LIVE/DEAD Sperm Viability Kit (Molecular Probes Inc., USA). The nucleic acid staining with SYBR-14 and propidium iodide (PI) was performed according to the manufacturer's instructions. Stained sperm cells were imaged using a fluorescence microscope with blue (460–490 ​nm) and green (530–550 ​nm) excitations for SYBR-14 and PI. While SYBR-14 stains all cells in green, PI only enters cells and stains them red when membrane integrity is compromised. Thus, the ratio of red and green stained cells measures cell viability. A total of 3296 sperm cells were analysed.

### 2.8 Redox status of FND-exposed sperm cells

The CellROX Green assay (Invitrogen) determined the total ROS production in sperm cells according to the manufacturer's instructions. Sperm cells were seeded in flat bottom 96-well plates (6000 ​cells per well) and incubated with FNDs at concentrations 1, 5, 10 and 20 μg/mL for 4 ​h in both uncapacitating (mHTF) and capacitating (HTF) medium. Sperm cells were then incubated for 30 ​min at 37 ​°C with 5 ​μM CellROX Green reagent in the culture medium. Afterwards, the samples were rinsed with fresh medium and compared fluorescence intensity with a FLUOstar Omega Microplate Reader (BMG Labtech, The Netherlands) at excitation at 485 ​nm, and emission at 520 ​nm. Measurements were performed for both uncapacitated and capacitated cells. Menadione 100 μM incubated for 1 ​h at 37 ​°C was used as a positive control. The results from three independent repetitions were normalised over the negative control (cells untreated with FNDs) after subtraction of the background (medium without cells).

### 2.9 Statistical analysis

Statistical analysis was performed using Graph pad prism version 7. Data were treated with the two-way ANOVA and Tukey test. Significance was tested compared to the control samples and defined as ns p ​> ​0.05, ∗P ​≤ ​0.05, ∗∗P ​≤ ​0.01, ∗∗∗P ​≤ ​0.001, ∗∗∗∗P ​≤ ​0.0001.

## 3 Results and discussion

### 3.1 The location of FNDs

**Fig. 1:**
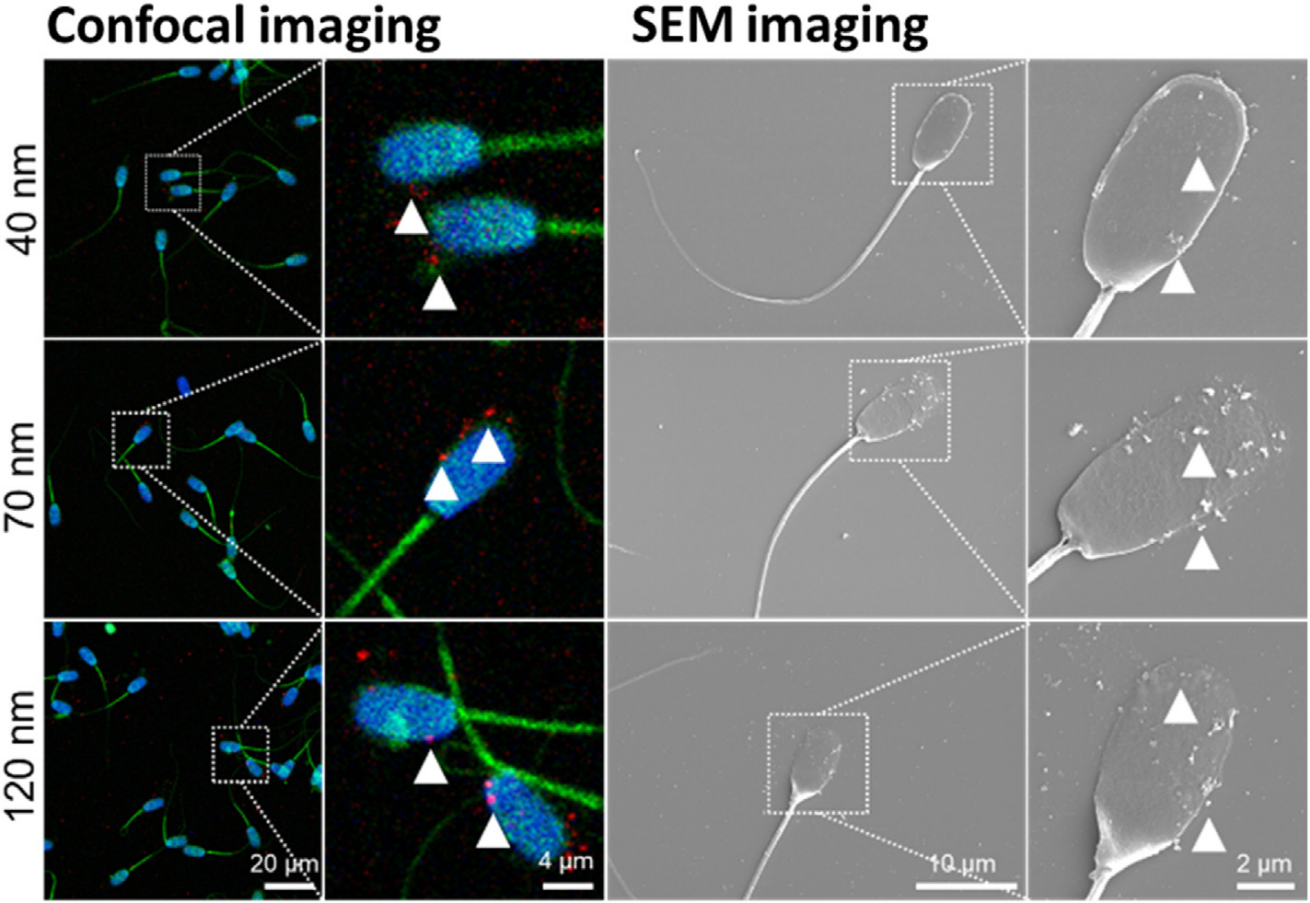
Distribution of FNDs in capacitated sperm cells. Distribution of 4 ​h incubation FNDs (1 ​μg/ml) attached to capacitated sperm cells immobilised on fibronectin measured by confocal microscopy (a) and SEM (b). The CLSM images presented the following structures: nucleus (blue), F-actin (green), FNDs (red). White arrows indicate FNDs attached to the head of sperm cells.

To understand FNDs interaction with sperm cells, we have investigated the distribution of particles using DLS (see Fig. S1), fluorescent confocal and scanning electron microscopy (see Fig. 1, Figs. S2 and S3). Due to the albumin protection, FNDs dispersed in the HTF medium have less aggregation than in the mHTF medium. We have observed fewer particles at the surface of cells treated with 40 ​nm FNDs than sperm cells treated with 70 ​nm or 120 ​nm FND variants. We have shown that 70 ​nm and 120 ​nm FNDs formed aggregates that were easier to visualise based on their fluorescent properties. The FNDs attach to the cells without a surface modification, making them easy to apply as sperm cells label.

### 3.2 Metabolic activity of FNDs-treated sperm cells

**Fig. 2:**
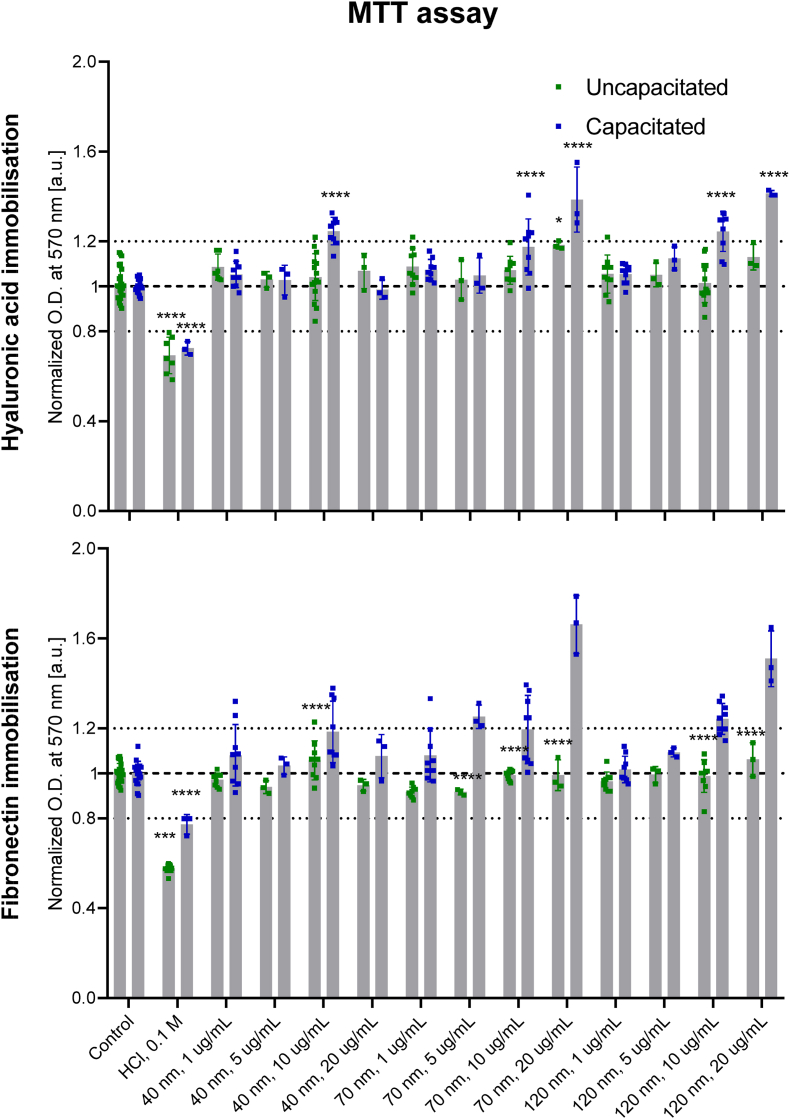
The metabolic activity of the sperm cells was immobilised on fibronectin or hyaluronic acid and treated with FNDs of various sizes and concentrations for 4 ​h before or after capacitation. Three independent experiments were performed. 0.1 ​M HCl was used as a positive control. The data was normalised against the negative control of sperm cells without treatment (line at 1).

The MTT assay measures the mitochondrial metabolic rate and indirectly reflects the cells' viability. Mitochondrial dehydrogenases reduce the tetrazolium salt MTT to water-insoluble purple formazan crystals. We used the MTT assay to evaluate the sperm's metabolic status in this study. In Fig. 2, we show that the coating used for spermatozoa immobilisation has not reduced the metabolic activity. Furthermore, the size and concentration of fluorescent nanodiamonds did not impact the mitochondria performance of capacitated sperm cells.

Similarly, uncapacitated sperm cells were not affected by the FND's size. Nevertheless, we have observed that the higher concentration of particles (10 ​μg/ml) slightly increased the metabolic activity of uncapacitated spermatozoa compared to untreated cells. It may suggest that FNDs induced hyperactivation (complex II) of mitochondria, leads to more dehydrogenase activity in sperm cells and higher conversion of MTT to formazan. Yazdanshenas et al. have also shown that supplementation of semen extender with zinc nano-complex improved mitochondrial activity of uncapacitated sperm [36]. Aminzadeh et al. have not observed any change in metabolic activity of uncapacitated spermatozoa after incubation with carbon nanotubes [37].

On the other hand, it has been shown that many types of nanoparticles decrease the metabolic activity of sperm cells. Wang et al. reported that silica NPs have an adverse effect on sperm cells of various maturity stages by damaging mitochondria's structure, resulting in energy metabolism dysfunction [38]. Other researchers have shown an adverse, dose-dependent effect of silver nanoparticles on the metabolic activity of sperm cells [39,40]. The NPs were internalised into the head and mitochondrial region of capacitated spermatozoa. It has been found that silver NPs alter the typical mitochondrial architecture and reduce mitochondrial activity due to disruption of the respiratory chain [41].

### 3.3 Effect of FNDs on sperm membrane integrity

**Fig. 3:**
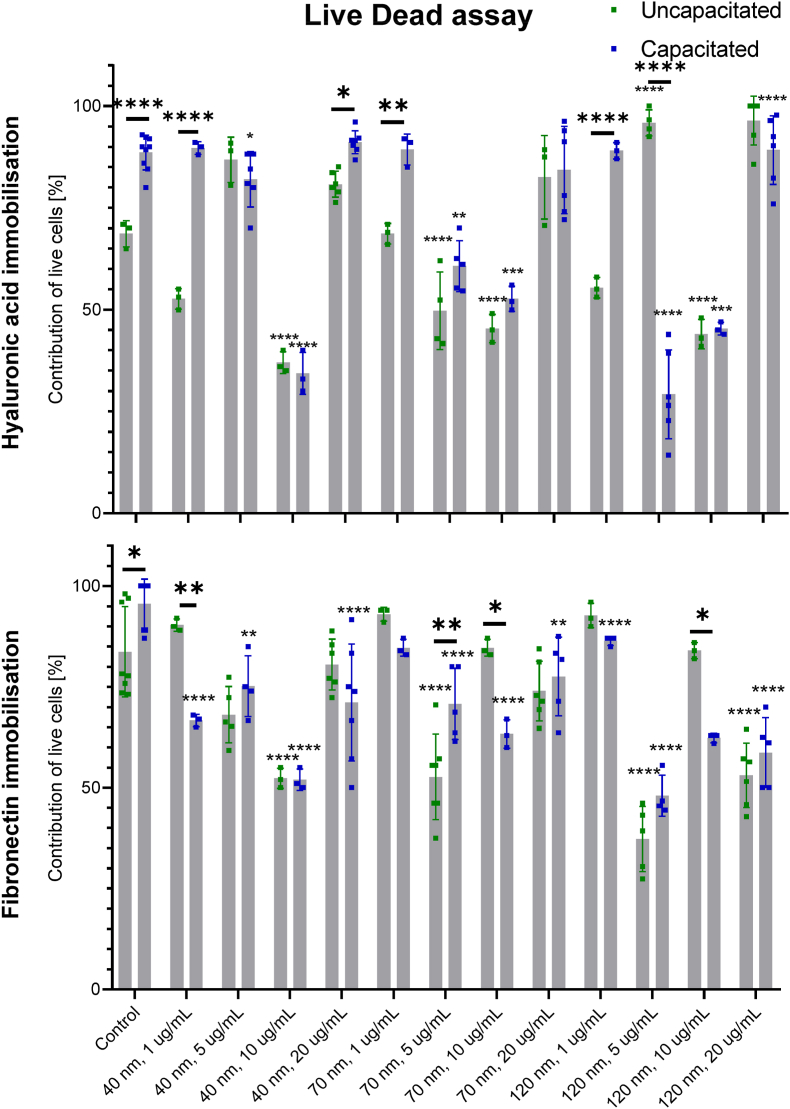
Membrane integrity of sperm cells immobilised on hyaluronic acid or fibronectin and treated with FNDs for 4 ​h before or after capacitation (gridded bars). Three independent experiments were performed. Negative control (grey bars) represent. Light colour bars (light blue, light purple and light pink) represent 1 ​μg/mL, while darker bars (dark blue, dark purple and dark pink) represent 10 ​μg/mL.

An interaction of NPs with the plasma membrane of sperm cells may lead to its reorganisation, disintegration, and, consequently, cell death. We show in Fig. 3 and S4 that the viability of spermatozoa depends on the size and concentration of FNDs. We further investigated immobilisation with fibronecting and hyaluronic acid. While we see some differences in membrane integrity, we do not see any clear trends or concentration dependencies. In some of the conditions we see a slight tendency that small concentrations are tolerated better than large ones. This is especially the case for the smallest 40 ​nm particles. 40 ​nm, 70 ​nm and 120 ​nm FNDs had a relatively low impact on sperm viability at 1 ​μg/ml. Based on our results, we can conclude that the impact of nanoparticles on the membrane integrity of sperm cells is dependent on the capacitation status. Sperm capacitation leads to increased plasma membrane fluidity and shifts its surface charge towards positive values. Therefore, the membrane of capacitated sperm cells facilitates the attachment of the negatively charged FNDs. The viability of cells after treatment with 40 ​nm FNDs was significantly lower for capacitated sperm cells than uncapacitated ones. We think this may also be related to the increased plasma membrane fluidity of the capacitated spermatozoa. Yoisungnern et al. showed that silver NPs stayed at the membrane when applied at low concentrations. Higher dosages were internalised into the spermatozoa, which resulted in membrane damage and decreased sperm viability [41].

In most group we observed more viable cells on the surface functionalised with fibronectin than with hyaluronic acid. This effect is likely not due to either of the coatings being toxic but ue to active and mobile cells being catured with different effectiveness. The comparison between fibronectin and hyaluronic acid has not been the subject of any sperm studies. So far, researchers have reported that sperm that attach to hyaluronic acid exhibit increased viability, maturity, acrosome integrity and reduced DNA fragmentation compared to a control [42]. Frimat et al. used fibronectin for individual trapping of sperm cells for analysis and recovery using micro-contact printing. They have not observed any loss of spermatozoa viability [43]. Further, it has to be noted, that an increase in membrane permeablity does not necessarily mean that cells are dead. Especially for capacitated sperm cells this has been reported before [44].

### 3.4 Redox status of FND-exposed sperm cells

**Fig. 4:**
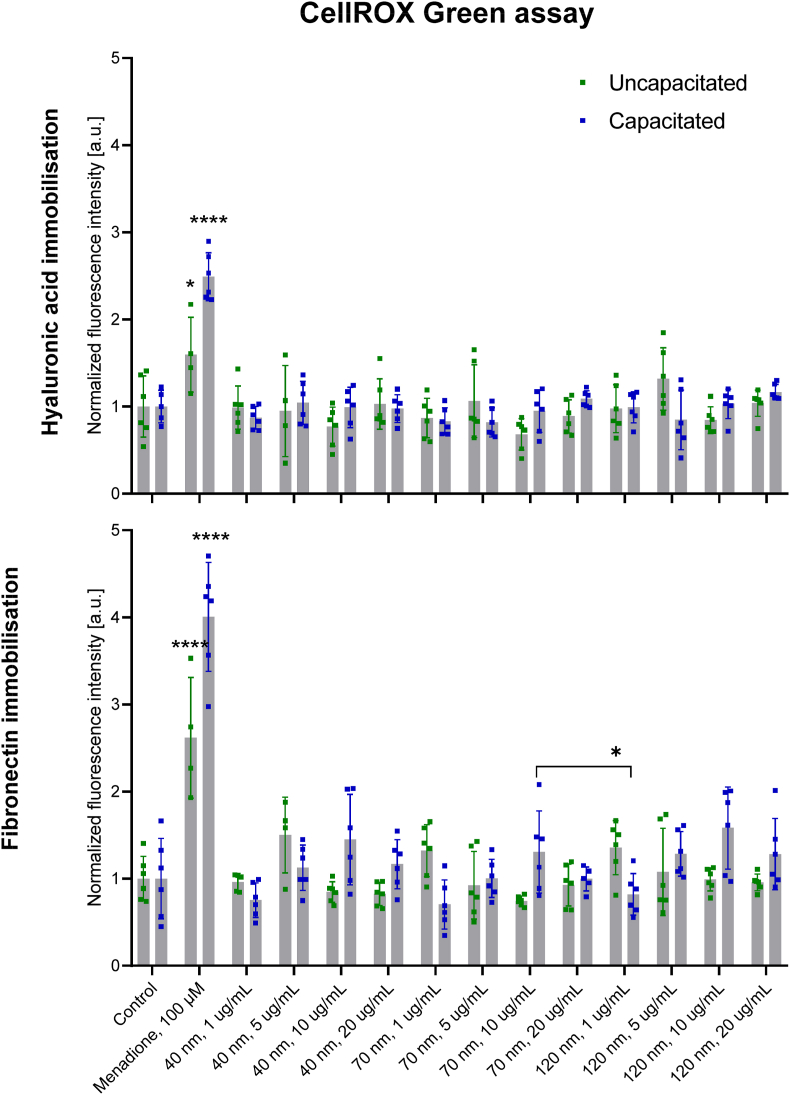
Redox status of sperm cells immobilised on fibronectin or hyaluronic acid and treated with FNDs of various sizes and concentrations for 4 ​h before and after capacitation. Three independent experiments were performed for each condition.

The redox status of a sperm cell is directly correlated with its potential for fertilisation. It has been shown that nanoparticles may trigger ROS generation [45]. Therefore, as part of the biocompatibility evaluation, we investigated the effect of FNDs on the redox status of spermatozoa. The results are shown in Fig. 4. We found that the concentration of FNDs had a significant effect on the ROS level in the uncapacitated sperm cells on the hyaluronic acid and fibronectin coating. There was a significant increase in ROS level in response to higher FNDs concentration (10 ​μg/ml), independently of the particle size. At the same time, a small effect was found in 40 ​nm and 120 ​nm FNDs at lower concentrations (1 ​μg/ml) for cells on fibronectin. We noticed that after capacitation, there is no clear concentration dependency of the redox status of spermatozoa. Further we didn't observe any differences when comparing different sizes of FNDs or the type of coating that we used to immobilise the sperm cells. Similarly, Yoisungnern et al., in their studies on silver nanoparticles, have presented elevated ROS generation as closely associated with a reduction in sperm viability and abnormal sperm morphologies.

## 4 Conclusions

Here we investigated the biocompatibility of nanodiamonds on sperm cells. We found that FNDs are not universally biocompatible for these cells as reported for other cell types. We found that cells tolerated larger particles better than the smallest particles and lower concentrations better than higher ones. Furthermore, biocompatibility depends on the developmental stage of the sperm. Mostly, uncapacitated cells were more resilient to stress induced by FNDs. Thus, different applications concerning nanodiamonds in the sperm cells must carefully choose the conditions to avoid introducing stress to the cells. Despite the fact that nanodiamonds are not universally biocompatible, we were able to confirm that for particles of 70 ​nm as well as 120 ​nm 1 ​μg/mL is a concentration that can be used without influencing the metabolic activity or cells viability. While this is a relatively low concentration for labelling, sensing metabolic activity in sperm cells is a promising application.

### Author contributions

CR-SM, YZh and AM conducted the experiments performed in this article with the help of TH, RL and AS. JK performed SEM imaging. LB and CK helped with data analysis. RS leads the research group, and RS and AM supervised this project. All authors have contributed to writing and editing and approved the article's final version.

## Declaration of competing interest

The authors declare the following financial interests/personal relationships which may be considered as potential competing interests:Romana Schirhagl reports financial support was provided by 10.13039/501100000780European Commission. Yue Zhang, Runrun Li reports financial support was provided by 10.13039/501100004543Chinese scholarship council.

## Data Availability

Data will be made available on request.
