# Supplementary material for: Fluorescent nanodiamond labels: Size and concentration matters for sperm cell viability
Source: Mater Today Bio. 2023 Apr 10;20:100629. doi: 10.1016/j.mtbio.2023.100629 (PMC10333662; doi:10.1016/j.mtbio.2023.100629)
Supplement: Multimedia component 1 [file mmc1.docx]

**Supplementary Information**


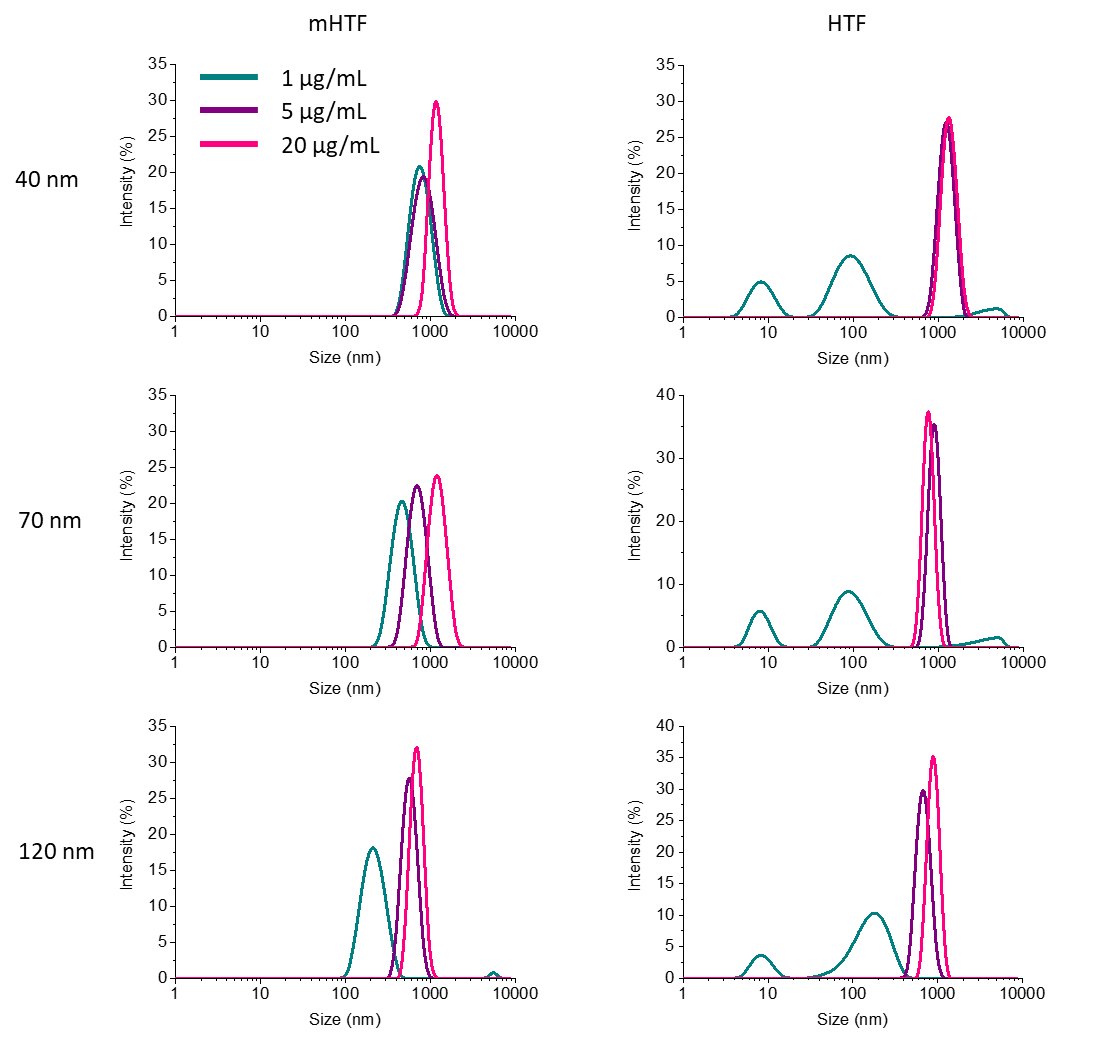


Figure S1. Size distribution of FNDs in uncapacitating (mHTF) and capacitating (HTF) media. FNDs at concentrations 1, 5 and 20 µg/mL were tested. All measurements were performed at 25 ^o^ C. NA means media only.


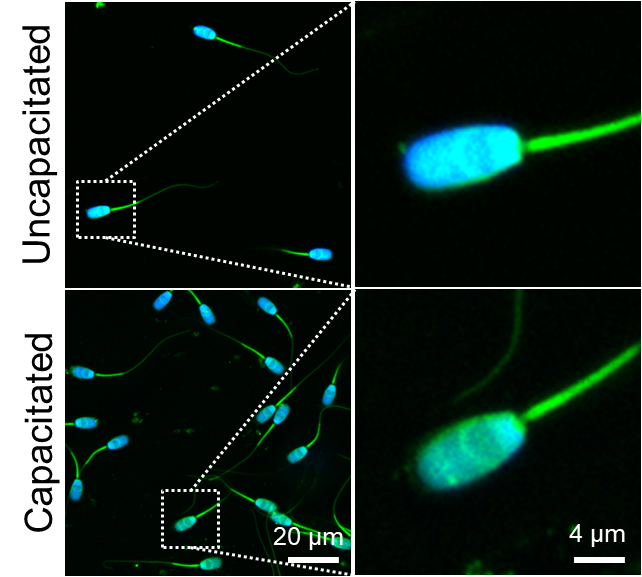


Figure S2. Capacitation status of sperm cells immobilised on fibronectin. The following structures were presented: blue – nucleus, green - F-actin. Sperm cells without FNDs treatment. Sperm cells under uncapacitating conditions (incubated in mHTF medium) show a balanced distribution of F-actin. At the same time, F-actin accumulates on the medial part of capacitated sperm cells (incubated on HTF medium).

**
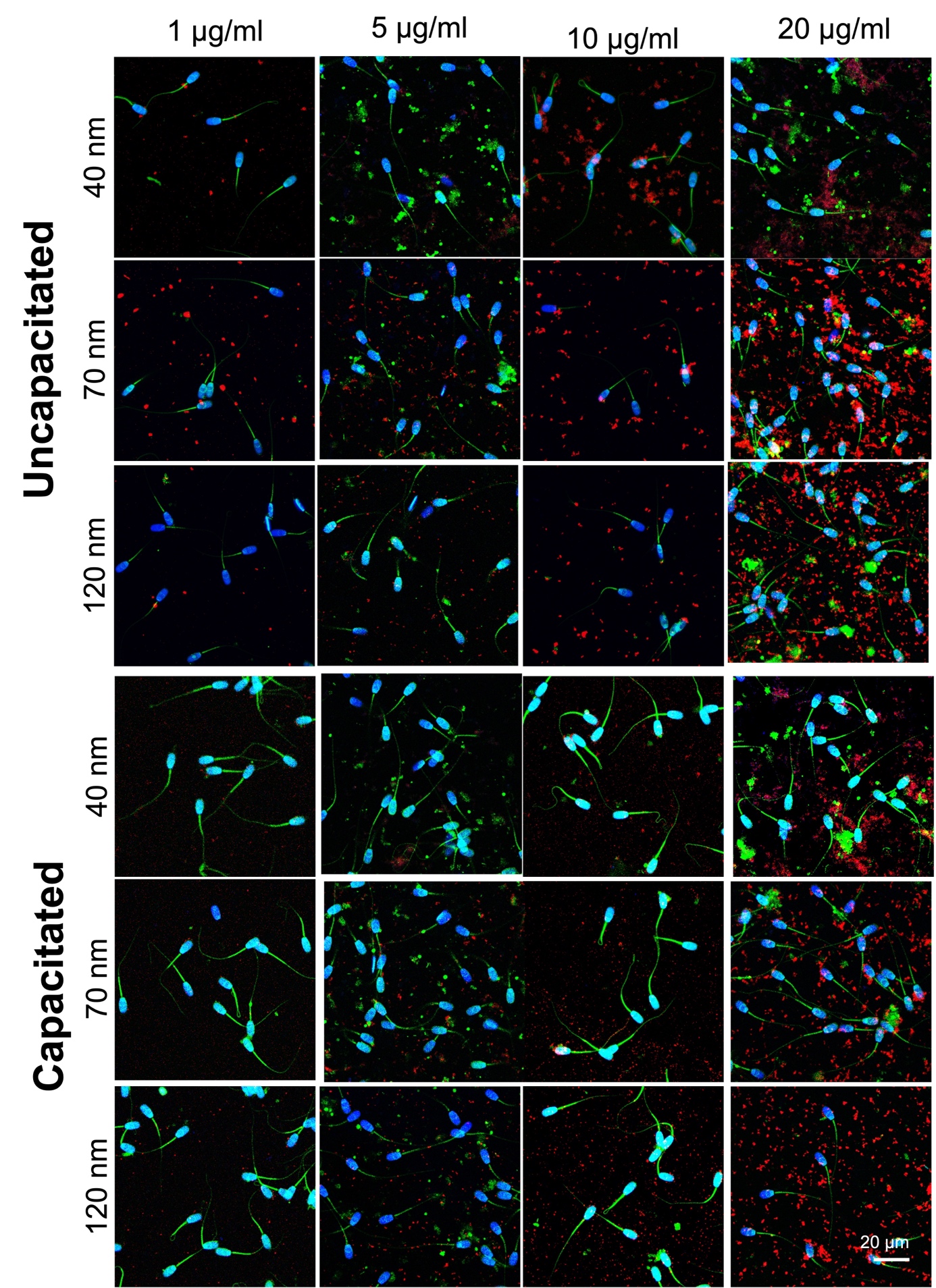
**

Fig. S3. Distribution of FNDs (1, 5, 10 and 20 µg/ml) attached to uncapacitated and capacitated sperm cells immobilised on fibronectin measured by confocal microscopy. The following structures were presented: blue – nucleus, green - F-actin, red - FNDs. White arrows indicate FNDs attached to sperm cells.


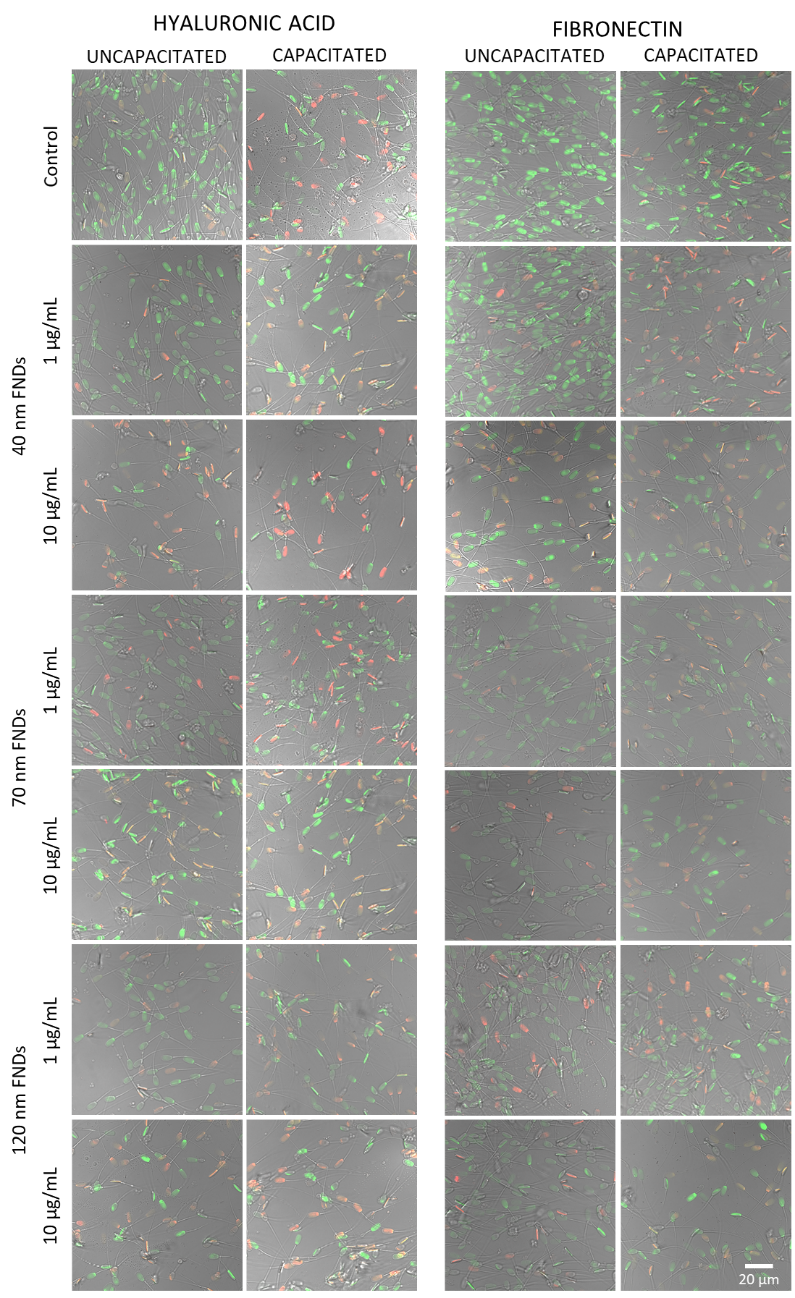


Figure S4. Membrane integrity of sperm cells immobilised on hyaluronic acid or fibronectin and treated with FNDs before and after capacitation. The brightfield and fluorescence images indicate sperm cells with integral membrane stained with SYBR-14 (green) sperm cells with membrane disrupted stained with propidium iodide (red). Scale bar 20 µm.





Figure S5. Particle characterization. To chemically characterize the particles, we have performed IR spectroscopy measurements. As expected we found below 3500 cm-1 which is characteristic for OH groups. Further, the C=O peak at 1700cm^-1^ is visible in all spectra.
